# The RNA-Binding Protein KSRP Is a Negative Regulator of Innate Immunity

**DOI:** 10.3390/biom16010030

**Published:** 2025-12-24

**Authors:** Vanessa Bolduan, Andrea Pautz, Matthias Bros

**Affiliations:** 1 Department of Dermatology, Medical Center of the Johannes Gutenberg University, 55131 Mainz, Germany; 2 Department of Pharmacology, Medical Center of the Johannes Gutenberg University, 55131 Mainz, Germany; pautz@uni-mainz.de

**Keywords:** KSRP, RNA-binding protein, innate immunity

## Abstract

KSRP (KH-type splicing regulatory protein) has emerged as a pivotal regulator of gene expression at multiple levels, acting as a transcription and splicing factor in the nucleus, and mediating AU-rich element (ARE)-dependent mRNA decay, translational silencing, and microRNA (miRNA) maturation in the cytoplasm. We and others have shown that KSRP acts as a regulator of immune responses, e.g., by dampening the expression of proinflammatory cytokines such as TNF-α, IL-6, IL-8, but also of NOS2, and facilitating the maturation of regulatory miRNAs, including let-7a, miR-129, and miR-155. This review aims to present current knowledge on the regulation of KSRP activity as conferred by miRNAs, phosphorylation, ubiquitination, SUMOylation, and interactions with long non-coding RNAs to enable dynamic responses towards inflammatory stimuli, and the effects of KSRP on innate immune reactions. Here, KSRP acts as an inhibitor by attenuating RIG-I-mediated antiviral signaling, cytokine production, and phagocytosis. In vivo, KSRP deficiency reduced arthritis severity but heightened inflammatory responses in sepsis and enhanced pathogen clearance in invasive pulmonary aspergillosis. These findings position KSRP as a dual regulator that limits tissue damage while constraining antimicrobial immunity. As a perspective, modulation of KSRP activity by applying pharmacological inhibitors may provide strategies to either suppress hyperinflammation in autoimmunity and sepsis or enhance host defense in immunocompromised states.

## 1. Introduction

The RNA-binding protein (RBP) KSRP (K homology [KH]-type splicing protein) has been identified by us and others as a negative regulator of inflammatory immune responses, by dampening the production of inflammatory mediators but also effector functions like phagocytosis and ROS (reactive oxygen species) production. This review intends to summarize the current knowledge on KSRP regulation and its role in regulating innate immune responses.

## 2. KH-Type Splicing Regulatory Protein (KSRP) Exerts Various Functions

**Figure 1:**
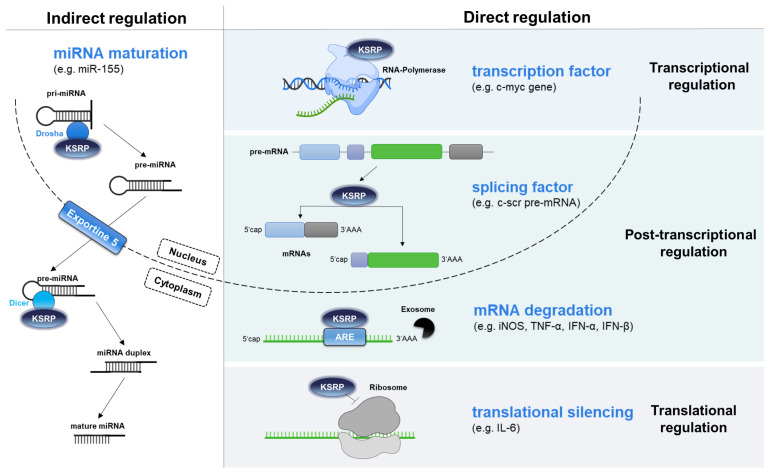
KSRP controls gene expression at multiple levels. It acts as a transcription and splicing factor in the nucleus and confers rapid degradation of ARE-containing mRNA in the cytoplasm and inhibits the translation of mRNA. Additionally, KSRP supports miRNA maturation by interacting with DROSHA and DICER (own illustration inspired by [10,11]).

KSRP (K homology [KH]-type splicing protein) regulates gene expression on multiple levels [1,2] (Figure 1). It acts as a transcription and splicing factor, but controls post-transcriptional gene expression as well [3]. This is achieved by promoting mRNA decay [4,5] and by inhibiting translation [6]. In both cases KSRP binds to an AU-rich element (ARE) that is located in its target mRNA. Moreover, KSRP also facilitates the maturation of miRNA species that subsequently inhibit gene expression [7,8,9]. Some of these regulatory mechanisms are described in more detail below.

KSRP has been shown to decrease the half-life of mRNAs encoding TNF-α, IL-8, type I and III IFN [1,2,5] and NOS2 [4] by binding to AREs [12]. KSRP appears to be a central constituent of ARE-mediated mRNA decay (AMD) [13,14]. Further, Lellek and colleagues identified KSRP as a component of the apolipoprotein B mRNA-editing enzyme-complex [15]. Winzen and coworkers identified about one hundred KSRP targets, including IL-6, IL-8, and Cyclooxygenase-2 mRNA that were upregulated in HeLa cells transfected with KSRP siRNA [16]. However, KSRP-dependent mRNA degradation was observed in only 10% of all target mRNAs, suggesting additional mechanisms of KSRP-mediated gene regulation. Furthermore, KSRP exerts influence not only on mRNA stability but may also attenuate mRNA translation, thereby impairing the expression of genes that encode pro-inflammatory cytokines and chemokines. Dhamija and colleagues investigated the polysomes of cells treated with KSRP-specific siRNA and observed that KSRP-deficient cells exhibited elevated IL-6 protein levels [6]. KSRP was demonstrated to interact with the AREs of IL-6 mRNA, thereby mediating its translational silencing.

The overall significance of RBPs as regulators of mRNA stability is also evident in the pathogenesis of inflammatory diseases. For instance, Tristetraprolin (TTP)-deficient mice spontaneously develop a chronic inflammatory phenotype, which has been attributed to the increased stability of mRNA species that encode pro-inflammatory mediators, including TNF-α [17], GM-CSF [18], and IL-6 [19,20]. Additionally, increased expression of pro-inflammatory genes has been observed in ARE/poly(U)-binding degradation factor (AUF)1-deficient mice [21]. Moreover, the elevated expression of numerous genes in inflammatory disorders has been attributed to a disruption in mRNA stability [22,23].

KSRP has a pivotal role in the processing of a subset of miRNAs with a GC-rich stem-loop in the precursor transcript [24]. The KH domain 3 of KSRP exhibits selective binding to G-rich sequences, thereby enabling KSRP to interact with the ribonucleases DROSHA and DICER. In the nucleus, KSRP facilitates the cleavage of pri-miRNA yielding pre-miRNA. Further, KSRP promotes the nuclear export of pre-miRNA by interacting with exportin-5. Further, KSRP confers the maturation of pre-miRNA by binding to the respective terminal loop, interacting with Dicer [13]. This process is essential for several miRNAs, including miR-155 [25], let-7a [26], and miR-129 [27], which are pivotal in regulating immune processes. Altogether, KSRP is a versatile RBP that regulates gene expression via mRNA decay and the maturation of miRNAs.

## 3. Regulation of KSRP Activity

### 3.1. mRNA Level

So far, transcriptional regulation of the KSRP gene remains largely unexplored; more detailed information about post-transcriptional regulation of KSRP is available. In this regard, the binding of miRNA-206 resulted in decreased KSRP mRNA levels in a myoblast cell line [28] (Table 1). In mammary gland cells, miRNA-27b-3p promoted KSRP mRNA decay [29]. Accordingly, KSRP silencing was a prerequisite for EMT transition in these cells [30]. In addition, Zhou et al. demonstrated that in *Cryptosporidium parvum*-infected gut epithelial cells, KSRP mRNA is translationally silenced by miRNA-27b-3p, resulting in enhanced NOS2 mRNA stability. The interaction of the RBP HuR with the ARE of KSRP mRNA results in stabilization of KSRP mRNA [31]. Hence, a knockdown of HuR resulted in reduced mRNA levels of KSRP.

### 3.2. Protein Level

**Figure 2:**
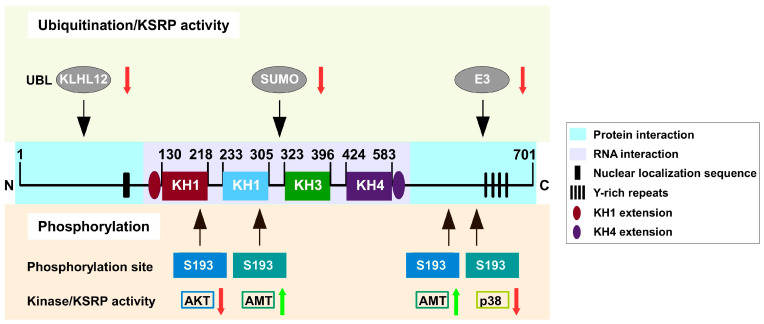
Modification of KSRP by phosphorylation and ubiquitination. AMT = ataxia telangiectasia mutated kinase; KLHL12 = Kelch-like protein 12; MAPK = mitogen-activated protein kinase; PKB = protein kinase B; SUMO = small ubiquitin-like modifier.

KSRP activity is regulated by phosphorylation and ubiquitination (Figure 2) as well as interaction with long non-coding RNAs (lncRNAs) (Table 2), as outlined in the following:

#### 3.2.1. Phosphorylation

KSRP has several phosphorylation sites that are targeted by several kinases, including p38 mitogen-activated protein kinase (MAPK) [32], protein kinase B (PKB) [33], and ataxia telangiectasia mutated (ATM) kinase [34], which in turn regulate KSRP activity [14]. Phosphorylation of KSRP by PKB on serine 193 stimulated the unfolding of unstable KH1, which yielded a binding site for 14-3-3-ζ [35]. The interaction of KSRP with 14-3-3-ζ resulted in nuclear accumulation of KSRP and accordingly reduced KSRP-dependent mRNA destabilization. Further, PKB-mediated phosphorylation prevented the association of KSRP with the exosome [33]. Additionally, DNA-activated ATM kinase was shown to phosphorylate KSRP at serine 274 and serine 670, respectively, thereby increasing the interaction of KSRP with pre-miRNAs, leading to increased biogenesis of KSRP-dependently processed miRNAs [2,36]. p38 MAPK phosphorylated KSRP at threonine 692 and inhibited its binding to ARE-containing target mRNA species, thus leading to a 2–3-fold increase in the stability of, e.g., myogenic transcripts encoding myogenin and protein 21 [32], as well as to an IL-1β-dependent increase in IL-8 expression [16]. KSRP inhibits prothrombin expression by binding to the upstream sequence element (USE) located in the 3′-UTR of the mRNA [37]. Upon phosphorylation by p38 MAPK, KSRP dissociates from the USE, resulting in increased mRNA stabilization. Similarly, KSRP-dependent silencing of IL-6 could be reversed by application of the pro-inflammatory cytokine IL-1 [6]. Here, the activation of p38 MAPK by IL-1 signaling resulted in KSRP phosphorylation, which reduced its binding to IL-6 mRNA.

Moreover, resveratrol has been shown to increase KSRP activity by inhibiting p38 MAPK-mediated phosphorylation at threonine 692 [38]. This resulted in increased degradation of various mRNAs encoding pro-inflammatory mediators, most probably contributing to the anti-inflammatory properties of resveratrol. Additionally, resveratrol has been demonstrated to impede TGF-β induced EMT in mammary gland cells, shown to depend on KSRP [39]. Altogether, the phosphorylation of KSRP at specific sites results in a reduction in KSRP activity, which in turn leads to elevated expression of immuno-relevant cytokines.

#### 3.2.2. Ubiquitination and SUMOylation

Ubiquitination is important for protein degradation and kinase activation, respectively. Ubiquitin can be conjugated to a protein as a single or multiple units (e.g., polyubiquitination) [40]. Ubiquitination of KSRP [41] plays a critical role in the course of viral infections. KSRP is involved in enterovirus internal ribosome entry site (IRES)-driven translation through modification of IRES trans-acting factors (ITAFs) [42,43]. In vitro experiments have demonstrated that KSRP activity is impaired through interaction with Kelch-like protein (KLHL)12. KLHL12 promotes polyubiquitination of KSRP at lysine residues 109, 121 and 122 in the C-terminal part of KSRP via linking to the cullin 3-based ubiquitin-protein E3 ligase complex. This leads to suppressed EV71 translation and favors the modulation of positively acting ITAFs [43].

Moreover, KSRP activity was observed to be affected by ubiquitination through small ubiquitin-like modifier (SUMO) in human embryonic kidney and prostate cancer cells [44]. SUMOs regulate target proteins by affecting their activity, stability, and localization [45]. SUMOylation of KSRP increased its accumulation in the cytoplasm, and at the same time resulted in the attenuated maturation of KSRP target miRNAs with G-rich stretches in their terminal loop (e.g., let-7 miRNA family) due to the disassociation of KSRP from the pri-miRNA/DROSHA complex. Moreover, KSRP seems to be a substrate of a multi-protein E3 ubiquitin ligase complex, containing F-box and WD repeat domain-containing 2 (FBXW2), S-phase kinase-associated protein 1 and cullin-1 F-box protein (SCF). Wang and coworkers demonstrated that, in murine macrophages, FBXW2 directly engaged KSRP and triggered its ubiquitination by SCF, leading to the degradation of KSRP [46]. To sum up, the ubiquitination of KSRP appears to cause attenuation in KSRP activity.

#### 3.2.3. Long Non-Coding RNAs

Long non-coding RNAs (lncRNA) are defined as >200 nt RNA species that are not translated into functional proteins [47]. SRP activity in the cytoplasm was reported to be modified by interaction with the lncRNA H19 [48]. In mesenchymal cells, this lncRNA engaged KSRP, which favored KSRP-mediated destabilization of labile transcripts, such as myogenin mRNA in C2C12 cells [48]. Upon PKB activation, KSRP was released from H19, resulting in enhanced stabilization of myogenin mRNA, but also elevated maturation of myogenic miRNAs, thus favoring myogenic differentiation. Furthermore, in epithelial tissues, KSRP activity was modified by interaction with the lncRNA Epithelial Program Regulator (EPR) [49]. This inhibited the KSRP-mediated decay of Cdkn1a mRNA, thus favoring cell proliferation. Furthermore, it has been demonstrated that axon-enriched long intergenic non-coding RNA regulating axon elongation (ALAE) interacts with KSRP and blocks its binding to its target GAP43 mRNA in neuronal cells [50]. The promotion of axon elongation by ALAE is achieved by increased translation of growth-associated protein 43 (Gap43) mRNA.

**Table 2:** Regulation of KSRP by lncRNAs.

lncRNA	Mechanism of Action	Functional Consequences	Ref.
H19	Sequesters KSRP, prevents its interaction with target mRNAs	Stabilization of *Myogenin* mRNA, thereby promoting skeletal muscle differentiation	[48]
EPR	Promotes proteasomal degradation of KSRP	Stabilization of *p21* mRNA, thereby inhibiting epithelial proliferation	[49]
ALAE	Sequesters KSRP, reduces its mRNA decay function	Stabilization of Gap43 mRNA in axons	[50]

In summary, KSRP activity is regulated on several levels, comprising transcriptional and post-transcriptional mechanisms.

## 4. KSRP as a Regulator of Innate Immune Responses

The innate immune system is the evolutionarily older arm of the immune system and is a complex network of initial responses to pathogenic organisms and environmental insults [51]. At the cellular level, the innate immune response is conferred by polymorphonuclear neutrophils (PMN), monocytes or macrophages (MAC), and dendritic cell (DC) populations [52]. These innate immune cells sense danger signals that are released in case of cell damage and/or infection by germline-encoded pattern recognition receptors like Toll-like receptors. The (RIG-)I-mediated antiviral pathway is an important component of the innate immune response to viral infections. RIG-I-like receptors recognize 5’-triphosphorylated RNA and activate a signaling cascade to induce type-I IFN-dependent responses [53]. KSRP was shown to inhibit RIG-I signaling by interacting with the regulatory domain of RIG-I, rendering the receptor inactive [54]. As a result, the antiviral response is attenuated. In agreement, when KSRP expression is reduced, RIG-I signaling becomes more active and this leads to a stronger antiviral response and reduced viral replication.

Regarding the differentiation of myeloid cell types, KSRP has been demonstrated to favor granulocyte development at the expense of monocyte differentiation by enhancing the maturation of miR-129 [55]. Further, in line with the central role of KSRP in AMD of, e.g., pro-inflammatory cytokines, KSRP has been recognized as a key regulator of inflammatory immune responses [5,56,57].

Based on these observations, we assessed the role of KSRP for innate immune responses in several disease models by comparing the respective course of disease in KSRP^-/-^ versus wild-type (WT) mice as presented in the following and summarized in Table 3.

### 4.1. CAIA

In our first study, we assessed the role of KSRP in a murine model of chronic joint inflammation, namely collagen antibody-induced arthritis (CAIA) [58]. KSRP^−/−^ mice developed only mild symptoms, whereas in WT mice, CAIA induction elicited robust joint inflammation that resembled progressive arthritis symptoms (Table 3). While WT mice presented with strong upregulation of KSRP-regulated transcripts (Cxcl-1, Nos2, Tnf-α) following CAIA induction, KSRP-deficient mice displayed less upregulation of either transcript. Histological examination showed much stronger immune cell infiltration into the joints of CAIA-treated WT mice. In line, KSRP^−/−^ mice presented with attenuated expression of lineage marker genes characteristic of myeloid cell types. Furthermore, the chemokine MCP-1, known to orchestrates myeloid cell recruitment, was less expressed in CAIA-treated KSRP^−/−^ mice. Altogether, these findings suggest that KSRP deficiency attenuates arthritis partly by impairing migration and accumulation of MAC and PMN that together orchestrate an immune response by regulating other immune cells as well as each other [59] in inflamed joints [58]. Likewise, the blood and spleen of CAIA-treated KSRP-deficient mice contained less myeloid cells than apparent in the respective WT control, without significant alterations in lymphoid cells. Moreover, we observed a higher frequency of apoptotic myeloid cells in the blood of KSRP-deficient CAIA-induced animals indicative of enhanced myeloid cell death contributing to attenuated infiltration of these cells into the joints.

Total spleen cells from untreated KSRP^−/−^ mice secreted higher levels of IFN-γ compared to WT in response to LPS stimulation, and the same trend was observed for spleen cells derived from CAIA-treated animals. Notably, no significant genotype-dependent differences were detected for TNF-α, IL-1β, or IL-10 production suggesting that while the loss of KSRP selectively reduces myeloid cell-mediated pro-inflammatory signaling in CAIA, it may concomitantly enhance IFN-γ responses, presumably via other immune cell populations.

### 4.2. KSRP Deficiency Led to Enhanced Generation of Pro-Inflammatory Cytokines in Sepsis

During sepsis, the innate immune system is activated by pathogen-derived molecular cues resulting in severe systemic inflammation, termed the cytokine storm [60]. Especially, IL-1β, along with IL-6 and TNF-α regulate early responses in sepsis progression [61]. The increased expression of these factors is, in part, due to enhanced activity of the required transcription factors (e.g., NF-κB, STATs, AP-1). However, genes that confer to inflammatory and immune responses are also subject to post-transcriptional regulation by RBP [62].

We demonstrated that KSRP-deficient mice exhibited elevated IL-1β levels in sera 12 h post-injection of LPS. In line, KSRP-deficient bone-marrow-derived MAC (BMDM) exhibited higher mRNA and protein expression of IL-1β following LPS stimulation [63]. Immunoprecipitation studies showed that KSRP directly binds to IL-1β mRNA in LPS-stimulated MAC. In contrast, according to these analyses, the regulation of IFN-α, IFN-β, IFN-γ, IL-6 and TNF-α mRNA by KSRP in MAC [63] and PMN [64] appeared to be indirect, occurring through mechanisms such as translational inhibition, as previously reported for IL-6 [6] or, e.g., via interaction with miRNAs [65]. Altogether, these findings suggest that KSRP limits the cytokine storm during sepsis [63]. Similarly, Liu et al. reported that the RBP AUF1 provided protection against sepsis by attenuating expression of pro-inflammatory cytokines, including IL-1β and TNF-α, via enhanced mRNA decay [21]. Mice with complete KSRP deficiency in all tissues have shown enhanced anti-viral responses upon infection, along with increased production of type I interferons [66], which is consistent with its key role in mRNA decay and its inhibitory effect on RIG signaling [54]. In line, we found in LPS-stimulated MAC from KSRP deficient mice that the genes upregulated most strongly are involved in regulating the innate immune response toward type I interferons [63].

### 4.3. KSRP Deficiency Inhibits IPA

Due to our finding that KSRP deficiency led to an increased inflammatory response by MAC in a sepsis model [63], we asked whether this could provide advantages in pathogen eradication. This hypothesis was evaluated in the model of invasive pulmonary aspergillosis (IPA), a major threat to immunocompromised individuals caused by the infection with the saprophytic fungus *Aspergillus fumigatus* [67]. In immunosuppressed patients, inhaled *A. fumigatus* conidia (AFC) can cause IPA, a potentially lethal disease that arises when conidia germinate in the lung and develop into hyphae [68]. The innate immune system, mainly PMN and MAC, are considered to constitute the primary line of defense conferring the clearance of conidia and preventing the growth of AFC [69,70,71].

Somewhat surprisingly, PMN-depleted KSRP^−/−^ mice survived inoculation with AFC, in contrast to the WT control group that died within the first three days [64], as previously observed [72]. Given that KSRP-deficient MAC showed stronger expression of pathogen defense-associated genes, which is in line with an enhanced pro-inflammatory response and exhibited increased phagocytic activity [63], it seems feasible to hypothesize that this immune cell population may compensate at least in part for depleted PMN. Further, we have previously reported that KSRP diminishes CXCL1-induced PMN migration [11]; CXCL1 constitutes the most important infection-induction chemoattractant for PMN [73]. Hence, increased migratory activity of KSRP^-/-^ PMN toward CXCL1 may explain in part the higher number of PMN in the bronchoalveolar lavage fluid and lungs of these mice.

In agreement with the hypothesis of elevated pathogen-killing activities of KSRP-deficient immune cells, including phagocytosis, degranulation, and the release of reactive oxygen species (ROS) [74], we observed that KSRP-deficient PMN and MAC showed higher phagocytic uptake of AFC. In addition, KSRP^-/-^ PMN produced significantly more levels of ROS. Moreover, KSRP^-/-^ PMN, which displayed an upregulation of genes linked to viability, showed attenuated apoptosis upon stimulation with LPS.

In addition, we observed that KSRP deficiency caused increased ROS production by PMN and that KSRP may regulate gene expression upon oxidative stress [64]. This effect may be mediated by protein kinases, such as p38 or PKB/Akt, that inhibit KSRP activity [75]. TTP, which is also known to regulate ROS-dependent mRNAs (e.g., GM-CSF, c-fos, Cox-2), was apparent at elevated levels in the liver and skeletal muscle in mice on a high-glucose diet, known to trigger inflammation and oxidative stress.

A considerable number of genes found to be increased in expression in stimulated KSRP-^-/-^ PMN were linked to cell viability [64]. In accordance, KSRP^-/-^ PMN were observed to exhibit diminished apoptosis, indicating that KSRP deficiency prolongs cell survival in this setting, which is in contrast to our findings in the CAIA model [58]. This may contribute to the increased frequency of PMN in the BAL/lungs of IPA-treated KSRP^-/-^ mice. In combination with elevated effector mechanisms, KSRP deficiency thereby may result in a notable enhancement of pathogen defense. As previously described by Ebner and colleagues, TTP-deficient PMN expressed more Mcl1 mRNA, a direct TTP target that encodes an anti-apoptotic protein relevant for PMN survival [76]. Thereby, similar to the results in KSRP-deficient mice, TTP deficiency reduces PMN apoptosis upon stimulation, whereas it is not impaired under steady state conditions.

In contrast to MAC that were characterized by upregulated IL-6 mRNA expression, PMN showed no genotype-dependent alterations, which underlines that KSRP may act in a cell-type-specific manner. Rather, KSRP^-/-^ PMN presented with elevated IL-6 production upon LPS stimulation, suggesting that KSRP not only affects mRNA stability but may also attenuate mRNA translation in a target mRNA-specific manner, thereby impairing gene expression. Furthermore, KSRP-deficient PMN demonstrated elevated levels of TNF-α mRNA and increased TNF-α secretion, while KSRP-deficient MAC exhibited higher mRNA levels of IFN-γ and in agreement, higher IFN-γ protein levels following LPS stimulation.

**Table 3:** Effects of KSRP deficiency on murine immune cell phenotypes and disease outcomes.

Disease Model	Immune Cell Types Examined	Immunophenotype Associated with KSRP Deficiency	Functional Effects and Disease Course (KSRP-Deficient vs. WT)	References
CAIA	Joint infiltrates; splenic myeloid cells	↓ expression of pro-inflammatory mediators in joints (CXCL1, NOS2, TNF-α, S100A8) ↓ immune cell infiltration ↓ splenic myeloid cell frequencies ↑ IFN-γ levels in splenic supernatants	Attenuated paw swelling Reduced joint inflammation Overall amelioration of arthritis severity	[58]
LPS (in vitro)	BMDM; peritoneal MAC	↑ induction of inflammatory and type I IFN-associated transcriptional programs ↑ cytokine expression (IL-1β, IL-6, TNF-α, CXCL1, CCL5)	-	[63]
	PMN	↑ ROS production upon stimulation ↑ activation-associated transcriptional signatures	-	[64]
Sepsis	Systemic cytokine response; MAC-centric	↑ systemic production of inflammatory cytokines	Exacerbated acute inflammatory response	[63]
*A. fumigatus* conidia (in vitro)	MAC, PMN	↑ phagocytic uptake of *A. fumigatus* conidia	-	[64]
IPA	PMN; MAC (lung, BALF)	↑ accumulation of PMNs and MACs in lung tissue improved survival compared with WT controls protection maintained independently of PMNs	Reduced pulmonary fungal burden Improved control of invasive fungal infection	

## 5. Perspectives

As an example for the outcome of RBP activity in innate immune cells on other tissues, inflammatory stimuli such as LPS have been shown to strongly enhance the expression of NOS-2 [4,77] through RBP-dependent post-transcriptional mechanisms and heme oxygenase-1 [78] particularly in MAC [79] and PMN [80]. The derived gaseous mediators NO and CO, respectively, act in a paracrine manner on endothelial and vascular smooth muscle cells, promoting inflammatory signaling, leading in turn to microcirculatory dysfunction [81] as shown in in vivo studies of endotoxemia in rat caudal artery reactivity models [82]. Further studies are required to study the functions of KSRP in other (innate) immune cells, including natural killer (NK) cells and other innate lymphoid cells (ILCs). This should encompass both homeostatic and stimulatory conditions in vitro, as well as relevant disease models. Especially concerning the potential role of KSRP for the regulation of type 2 cytokines, we recently showed that KSRP-deficiency leads to increased production of Th2-associated cytokines, resulting in exacerbated allergic asthma symptoms in vivo. It appears that KSRP primarily suppresses IL-4, thereby attenuating IL-13 expression [83].

So far, all studies regarding the role of KSRP in disease models have been performed using mice with a constitutive knockout of this protein. To delineate more specifically the cell-type-specific role of KSRP, mice with a conditional knockdown should be employed. To this end, mice with a floxed Ksrp gene locus may be crossed with mice expressing Cre recombinase under the control of the Cfsr1 (MAC), Ly6G (PMN), or CD4 (respective T cell population) promoter.

By now, also for some other RBP, an important regulatory role on innate immune cell function has been established. Regarding more recent publications, NK cell-specific deletion of Regnase-1 was reported to elevate their IFN-γ production by enhancing expression of the relevant transcriptional regulators such as OCT2 and IκBζ, resulting in the inhibition of tumor growth in vivo [84]. Further, in ILC2s, a mutant Regnase-1 resistant to IκB kinase-mediated degradation reduced proliferation in response to stimulation and diminished production of type-2 cytokines and increased cell death [85]. In agreement, such animals presented with attenuated type-2 pulmonary inflammation and helminth expulsion. Further, TTP was demonstrated to enhance antiviral innate immune signaling in a cell line by binding to RIG-I and promoting its K63-linked ubiquitination, thereby enhancing type I interferon responses during viral infection [86].

Regarding therapeutic perspectives, it may be possible to enhance KSRP activity to temporarily attenuate unwanted inflammatory immune responses, including sepsis, autoimmune diseases, or allergies. Conversely, the inhibition of KSRP may boost pathogen defense in patients with hampered immune system activity, as evidenced by the enhanced pathogen-killing activities of KSRP-deficient MAC and PMN. In conclusion, KSRP, on the one hand, serves to prevent excessive tissue damage in the host; however, on the other hand, it attenuates pathogen-killing activities. Further studies are required to better understand whether KSRP exerts its influence on immune responses exclusively through its action only on effector cytokines or if it also affects the expression of key transcription factors.

## 6. Conclusions

Taken together, the data presented herein demonstrate that KSRP plays a pivotal role in limiting anti-pathogen activity in PMN and MAC. Comparative gene expression analysis identified 64 genes that were commonly upregulated in KSRP^-/-^ PMN and MAC upon stimulation with LPS. A number of these were commonly involved in the positive regulation of anti-viral and anti-bacterial responses also comprising the IL-6 signaling pathway. However, the observation that most KSRP-regulated genes differed between both cell types underscores that KSRP acts cell-type-specifically. In general, RBP activity in MAC and PMN may also indirectly affect other compartments, such as for example vascular cells, since inflammatory stimuli strongly induce NOS-2 and heme oxygenase-1 via post-transcriptional mechanisms. The resulting mediators NO and CO act on vascular cells and promote inflammatory signaling, leading to microcirculatory dysfunction. The yet unknown role of KSRP in other innate immune cells, including NK cells and innate lymphoid cells, should be investigated under homeostatic and inflammatory conditions and in relevant disease models. So far, disease studies have relied on constitutive KSRP knockout mice. Future work should employ conditional, cell-type-specific approaches using appropriate Cre driver strains. From a therapeutic point of view, enhancing KSRP activity in MAC and PMN may limit pathological inflammation, whereas its inhibition could strengthen antimicrobial defense. Thus, KSRP appears to balance tissue protection and pathogen clearance, warranting further investigation into its effects beyond effector cytokines.

## Figures and Tables

**Table 1:** Regulation of KSRP mRNA by miRNAs.

miRNA	Mechanism	3′-UTR Binding Region/Gene Context	Biological Context	Reference
miR-206	Direct binding to multiple conserved sites	≥2 functional sites at ~nt 780–810 and ~1020–1050	Skeletal muscle differentiation; Duchenne model	[28]
miR-27b-3p	Direct/TGF-β-induced silencing	Binds KHSRP 3′UTR in NMuMg and MCF10A cells	TGF-β-induced EMT in mammary epithelial cells	[30]

## Data Availability

No new data were created or analyzed in this study.

## Abbreviations

The following abbreviations are used in this manuscript:

AFC	*A. fumigatus* conidia
ALAE	regulating axon elongation
AMD	ARE-mediated mRNA decay
AMT	ataxia telangiectasia mutated kinase
AP-1	Activator protein 1
APC	antigen-presenting cells
ARE	adenine-uracil-rich element
AUF1	ARE/poly(U)-binding degradation factor
CAIA	Collagen antibody-induced arthritis
CXCL1	C-X-C-motif ligand 1
DCs	Dendritic cells
EMT	epithelial-to-mesenchymal transition
EPR	Epithelial Program Regulator
GM-CSF	Granulocyte/Macrophage Colony-Stimulating Factor
HuR	human antigen R
IFN	Interferon
IL	interleukin
ILC	Innate lymphoid cell
iNos2	Inducible nitric oxide synthase
IPA	Invasive pulmonary aspergillosis
IRES	enterovirus internal ribosome entry site
ITAF	IRES trans-acting factor
KH	K homology
KLHL12	Kelch-like protein 12
KS(H)RP	KH-type splicing regulatory protein
lncRNA	Long non-coding RNA
LPS	lipopolysaccharide
MAPK	mitogen-activated protein kinase
miRNA	microRNA
mRNA	messenger RNA
NF-κB	nuclear factor ‘kappa-light-chain-enhancer’ of activated B-cells
NK	Natural killer
PKB	protein kinase B
PMN	polymorphonuclear neutrophils
RBP	RNA-binding protein
RIG-I	Retinoic acid-inducible gene I
ROS	Reactive oxygen species
SCF	S-phase kinase-associated protein 1 and cullin-1 F-box protein
STAT	Signal Transducers and Activators of Transcription
SUMO	small ubiquitin-like modifier
TGF-β	Transforming growth factor-β
TNF	tumor necrosis factor
TTP	Tristetraprolin
USE	Upstream sequence element
UTR	untranslated region
WT	Wild type
